# Identifying sub-health symptom clusters among urban older adults: exploring associations with Tai Chi exercise and risk of falling

**DOI:** 10.3389/fpubh.2026.1827783

**Published:** 2026-06-29

**Authors:** Tongtong Hao, Juntong Yuan, Ying He

**Affiliations:** China Wushu School, Beijing Sport University, Beijing, China

**Keywords:** fall risk, latent profile analysis, older adults, precision prevention, sub-health, Tai Chi

## Abstract

**Background:**

Sub-health status in urban older adults is multidimensional and heterogeneous, potentially influencing fall risk. This study identifies sub-health symptom clusters and explores how Tai Chi’s benefits vary across these groups.

**Methods:**

From December 2025 to February 2026, 612 sub-healthy older adults in Beijing were analyzed. Latent Profile Analysis (LPA) identified symptom patterns. Differences in demographics and fall risk indicators (fall rate and ABC scale) were compared across clusters. Stratified analysis assessed the association between Tai Chi exercise and fall risk within each cluster.

**Results:**

Three clusters were identified: Low Physical-Psychological (C1, 21.6%), Balanced Average (C2, 43.9%), and Cognitive-Social Advantage (C3, 34.5%). Significant demographic gradients existed across groups (all *p* < 0.001). Fall risk also showed a significant gradient: C1 had the highest fall rate (67.4%) and lowest ABC score (56.8 ± 9.6), while C3 had the lowest fall rate (7.6%) and highest ABC score (88.9 ± 6.5). Stratified analysis revealed that Tai Chi practitioners across all clusters had significantly lower fall rates and higher ABC scores than non-practitioners. Notably, C1 showed the greatest benefits, with the largest mean difference in ABC scores (14.6 points) and the most pronounced disparity in fall rates (12.2% vs. 92.3%).

**Conclusion:**

Sub-health status in urban older adults exhibits significant heterogeneity, with gradient differences in fall risk and Tai Chi benefits. Older adults in the Low Physical-Psychological group face the highest risk but derive the most significant gains from Tai Chi. Community health management should integrate sub-health profiling to develop personalized interventions. Prioritizing Tai Chi for health-impaired individuals can ensure precise resource allocation and maximize fall prevention outcomes.

## 1 Introduction

As the global population aging process accelerates, the health issues of older adults have received increasing attention. Falls are the leading cause of injury-related death in older adults aged 65 and above, with approximately one-third of community-dwelling older adults experiencing at least one fall annually (1). Falls not only lead to physical injury and functional impairment but also trigger a Fear of Falling (FOF), creating a vicious cycle of “fall—fear—reduced activity—functional decline—recurrent fall (2).” Therefore, identifying relevant factors for fall risk and developing effective prevention strategies is a critical task in the field of aging and public health.

Sub-health is an intermediate state between health and disease, manifesting as multidimensional functional decline across physical, psychological, and social adaptation domains (3). The prevalence of sub-health among urban older adults in China is high. Multidimensional symptoms—such as fatigue, lack of energy, decreased balance, and low mood—may have potential associations with fall risk (4). However, traditional variable-centered research treats older adults as a homogeneous group, ignoring the heterogeneity of intra-individual symptom combinations (5). In recent years, person-centered symptom cluster identification methods, such as Latent Profile Analysis (LPA), have been widely applied in aging health research. These methods can identify subgroups with distinct symptom characteristics, providing a basis for precision intervention (6, 7).

Tai Chi, a traditional mind–body exercise originating in China, has been proven by extensive research to effectively improve motor function and reduce fall risk in older adults (8). A recent network meta-analysis indicated that Tai Chi has a significant effect on improving fall efficacy in older adults and is most likely to be the optimal intervention measure (9). Its mechanisms of action include improving balance, enhancing muscle strength, optimizing gait strategies, and activating motor-related brain regions (10). However, existing studies mostly focus on the average intervention effect of Tai Chi, and few studies have explored the differences in benefits derived from Tai Chi exercise among older adults with different sub-health symptom clusters.

Based on this, the present study intends to use Latent Profile Analysis to identify heterogeneous symptom clusters of sub-health status in urban older adults, compare the differences in fall risk across different clusters, and explore the association between Tai Chi exercise and fall risk within each cluster. The results of this study can provide a basis for precision public health interventions and guide the development of personalized Tai Chi exercise recommendations based on the characteristics of different symptom clusters.

## 2 Methods

### 2.1 Research design

This study employed a cross-sectional survey design and utilized a multi-stage stratified random sampling method. The study is reported in accordance with the STROBE (Strengthening the Reporting of Observational Studies in Epidemiology) statement (11).

### 2.2 Participants

From December 2025 to February 2026, a survey was conducted in Beijing using a multi-stage stratified cluster sampling method (12). The sampling process aimed to obtain a representative sample of community-dwelling older adults to cover populations with diverse Tai Chi exercise habits. The process was divided into four stages:

Stage 1: Selection of Primary Sampling Units (PSUs) (13). Based on the density of the older adults population and the prevalence of Tai Chi activities across various districts in Beijing, a Probability Proportional to Size (PPS) sampling method was used. Three districts were randomly selected as PSUs from the central urban areas of Beijing (Dongcheng, Xicheng, Chaoyang, Fengtai, Shijingshan, and Haidian).

Stage 2: Selection of Secondary Sampling Units (SSUs). Within each selected district, four sub-district offices were randomly chosen from the available lists using simple random sampling, resulting in a total of 12 sub-districts (4 per district).

Stage 3: Determination of Survey Communities and Measurement Sites. Within each sub-district, 2–3 community committees were randomly selected as the basic survey units. Survey sites covered two types of locations: (1) Community public spaces: including community parks (e.g., Haidian Park, Yuan Dynasty City Wall Relics Park, Temple of Heaven Park), cultural squares, activity centers for older adults, and riverside fitness trails, to cover older adults with Tai Chi exercise habits; (2) Residential homes or community service centers: with the assistance of community committees, home visits or centralized surveys were conducted to ensure the inclusion of homebound older adults or those without regular exercise habits.

Stage 4: Systematic Sampling and Subject Recruitment. At each survey site, older adults were recruited using systematic sampling (e.g., sampling by house number intervals or intercepting at fixed intervals during morning exercise periods). A total of 1,705 valid questionnaire responses were obtained (effective response rate: 91.3%). Subsequently, the Sub-Health Measurement Scale Version 1.0 (SHMS V1.0) was used to screen participants and identify those in a sub-health state (14).

Inclusion and exclusion criteria:

*Inclusion criteria*: (1) Age ≥ 60 years; (2) Total SHMS V1.0 score below 60% of the maximum score (i.e., < 48 points), meeting the diagnostic criteria for sub-health status.

*Exclusion criteria*: (1) Severe cardiovascular or cerebrovascular diseases, mental disorders, or cognitive impairment; (2) Acute illness or trauma within the past 3 months; (3) Inability to complete the questionnaire independently.

**Figure 1:**
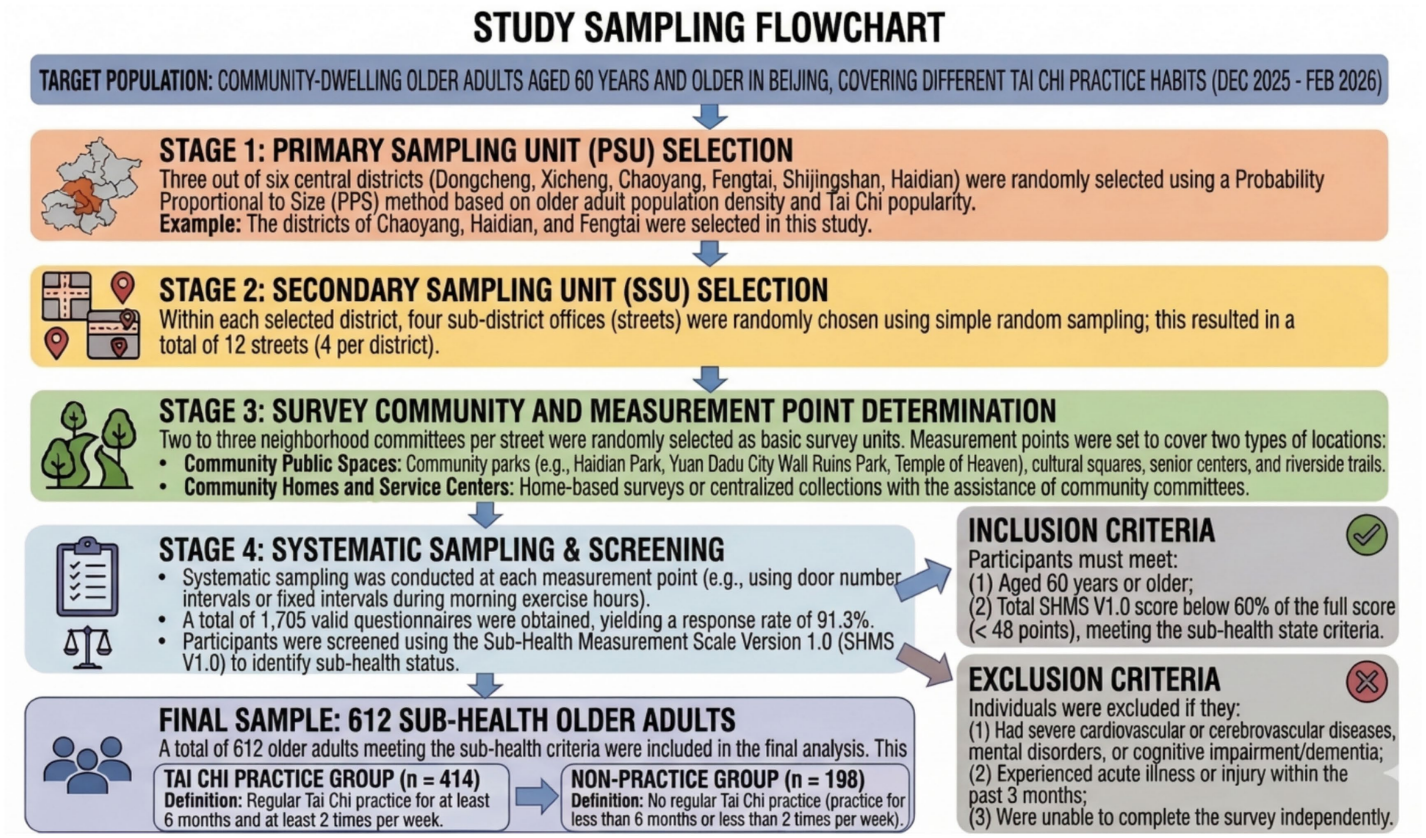
Sampling flowchart.

Ultimately, 612 older adults meeting the sub-health criteria were included in the subsequent Latent Profile Analysis and regression analysis, accounting for 36.25% of the valid respondents. Based on whether they practiced Tai Chi regularly (defined as continuous practice for ≥ 6 months, at least twice a week), participants were divided into two groups: the Tai Chi group (*n* = 414) and the Non-practitioner group (*n* = 198). The grouping process is detailed in Figure 1.

### 2.3 Sample size and ethics

The target sample size was determined *a priori* to satisfy the requirements of both Latent Profile Analysis (LPA) and Logistic regression simultaneously (15). For LPA: Methodological research suggests a minimum total sample of 300 to 500 cases, with at least 50 cases per latent profile to ensure stable parameter estimation (16). Given our aim to identify 3–5 profiles, a minimum of 250–300 sub-healthy participants was required. For Logistic Regression: Based on the Events Per Variable (EPV) principle, a sample size of 10–20 times the number of independent variables is recommended. With 10–15 variables and an estimated fall incidence of 20%, we aimed for a minimum of 500–750 cases. Adequacy of the Final Sample: After screening 1,705 respondents, the final analytical sample of 612 sub-healthy older adults was obtained. This sample size (*N* = 612) simultaneously satisfies both criteria: it meets the EPV requirement for regression and ensures that even our smallest identified profile (C1, *n* = 132) significantly exceeds the 50-case threshold for robust LPA.

The study protocol was approved by the Ethics Committee of Beijing Sport University (Approval No.: 2025347H) and was conducted in accordance with the ethical standards of the Declaration of Helsinki. All participants were informed of the study’s purpose and provided written informed consent prior to participation.

### 2.4 Instruments

#### 2.4.1 General information questionnaire

A self-designed general information questionnaire was used to collect sociodemographic information and health status (17). Sociodemographic variables included age, gender, education level, marital status, and monthly income. Additional health-related factors included Body Mass Index (BMI), smoking status, and alcohol consumption.

#### 2.4.2 Sub-health measurement scale version 1.0 (SHMS V1.0)

The Sub-Health Measurement Scale Version 1.0 (SHMS V1.0), developed by Xu Jun’s team, was used to assess the sub-health status of the participants (18). Developed based on the World Health Organization (WHO) definition of health and adapted to the Chinese cultural context, this scale has been validated with good reliability and validity among community-dwelling older adults in China (19). The scale consists of 39 items across three second-order dimensions and nine first-order dimensions: Physical Sub-health Subscale (14 items): Includes four dimensions: physical symptoms, organ function, motor function, and energy. Psychological Sub-health Subscale (12 items): Includes three dimensions: positive emotions, psychological symptoms, and cognitive function. Social Sub-health Subscale (9 items): Includes two dimensions: social adaptation and social support. The scale uses a 5-point Likert scale (1–5 points). The raw scores for each dimension are converted into a centesimal system using the following formula:

$\text{ConvertedScore}=\frac{\text{RawScore}-\text{TheoreticalMinimum}}{\text{TheoreticalMaximum}-\text{TheoreticalMinimum}}$ × 100

This centesimal conversion was applied at the dimension level for each of the nine sub-health dimensions. The converted scores range from 0 to 100, where higher scores indicate better health status. According to the diagnostic criteria, a total SHMS V1.0 score below 60% of the maximum (i.e., < 48 points) is defined as a sub-health state. In the present study, the Cronbach’s *α* coefficient for the total scale was 0.92, and the coefficients for individual dimensions ranged from 0.78 to 0.89, indicating good internal consistency reliability.

#### 2.4.3 Tai chi exercise questionnaire

A self-designed Tai Chi exercise questionnaire was used to collect information on the participants’ Tai Chi practice (20). The content of the questionnaire was determined based on a literature review and a preliminary pilot study. It consists of five core indicators measuring exercise frequency, duration, years of practice, exercise format, and Tai Chi style: Exercise Frequency: Measured by a single item: “How many times per week do you practice Tai Chi?” Options were set as the specific number of times per week (sessions/week). Exercise Duration: Measured by a single item: “What is the average duration of each Tai Chi session?” Options were set as the specific number of minutes (minutes/session). Years of Practice: Measured by a single item: “How long have you been continuously practicing Tai Chi?” Options were set as the specific number of years (years). Exercise Format: Measured by a single item: “In what format do you usually practice Tai Chi?” Options included group practice, individual practice, or a mixed format. Tai Chi Style: Measured by a single item: “Which Tai Chi style do you primarily practice?” Options included Yang style, Chen style, Simplified 24-form, or other styles. The pilot study showed that the test–retest reliability of the five items (with a 2-week interval) ranged from 0.75 to 0.88, indicating good measurement stability.

#### 2.4.4 Fall risk assessment tools

Fall risk was assessed using dual indicators to distinguish between psychological perception and actual outcomes. Fall history served as the objective outcome measure of past incidents, while the ABC Scale was employed to assess perceived balance confidence and fear of falling. Fall History in the Past Year: A single-item question was used: “Have you experienced a fall in the past year?” (0 = No, 1 = Yes). If the answer was “Yes,” further information regarding the number of falls and whether they resulted in injuries was collected. This indicator served as the objective outcome measure for fall risk (21). Activities-specific Balance Confidence Scale (ABC Scale): The Chinese version of the ABC Scale was used to assess the fall efficacy of older adults (22). The scale consists of 16 items that evaluate the level of confidence in not falling while performing daily activities (e.g., walking around the room, walking up and down stairs, walking on slippery surfaces). Each item is rated on a scale of 0 to 100 (0 = no confidence, 100 = complete confidence). The total score is the average of the 16 items, where higher scores indicate stronger balance confidence and lower fear of falling. In this study, the Cronbach’s *α* coefficient for the scale was 0.94, indicating excellent reliability.

### 2.5 Quality control

To ensure the scientific integrity and reliability of the data, quality control was implemented across three stages: investigator training, survey implementation, and data entry (23). Investigator Training: Prior to the survey, all research assistants underwent a standardized 3-day training program covering scale interpretation, interview techniques, and instrument operation. Only those who passed the final assessment were permitted to participate in the formal investigation (23). Survey Implementation: Field surveys were conducted during morning exercise periods (6,30–9,00 a.m.) at fixed booths set up in community parks and Tai Chi practice sites. Face-to-face interviews were employed. For participants with poor vision or comprehension difficulties, investigators read the items aloud to ensure full understanding. Questionnaires were collected on-site and checked for completeness; any missing items were supplemented immediately. Data Entry and Cleaning: Data were entered using EpiData 3.1 software with double-entry and consistency comparison (24). Any discrepancies were corrected by referring back to the original paper questionnaires. Before analysis, invalid questionnaires were excluded based on the following criteria: missing key variables, patterned responses (e.g., all answers identical), more than 20% missing items, or obvious logical errors. Bias Control: To minimize bias, all investigators used unified instructions, and all instruments were calibrated before use. Fall history was limited to the past 12 months to control for recall bias (25). Additionally, 10% of the participants were randomly selected for telephone follow-ups, and the consistency rate for key information exceeded 95%.

### 2.6 Statistical analysis

Data analysis was performed using SPSS 26.0 and Mplus 8.3 (26, 27). All statistical tests were two-sided, with the significance level set at *α* = 0.05. In descriptive statistics, continuous variables were presented as Mean ± Standard Deviation (SD), while categorical variables were expressed as frequencies and percentages (*n*, %).

Latent Profile Analysis (LPA): LPA was conducted using Mplus 8.3 to identify heterogeneous sub-health symptom clusters among urban older adults. The nine dimensions of sub-health (physical symptoms, organ function, motor function, energy, positive emotions, psychological symptoms, cognitive function, social adaptation, and social support) were used as external indicators. In the LPA, scores of these nine dimensions were treated as continuous indicators. The model assumed local independence within latent classes, with means and variances freely estimated for each class. Parameters were estimated using the maximum likelihood method. Model fit was evaluated using the Akaike Information Criterion (AIC), Bayesian Information Criterion (BIC), and sample size-adjusted BIC (aBIC), where smaller values indicate a better fit (28). The Lo–Mendell–Rubin Likelihood Ratio Test (LMRT) and Bootstrap Likelihood Ratio Test (BLRT) were used to compare the *k*-class model with the (*k*-1)-class model; a *p* < 0.05 indicated that the *k*-class model was significantly superior (29). Entropy was used to assess classification accuracy, with values > 0.80 indicating high precision. The optimal number of classes was determined by considering fit indices, classification quality, and theoretical interpretability.

Comparison of Group Differences: Chi-square tests were used to compare demographic characteristics and fall history across different latent profiles. Analysis of Variance (ANOVA) was employed to compare sub-health dimension scores and ABC scale scores among profiles. For significant differences, Bonferroni post-hoc tests were further conducted for multiple comparisons (30).

Stratified Analysis: To explore the association between Tai Chi exercise and fall risk within each cluster, stratified analysis was performed by splitting the dataset based on latent profile categories. Independent samples *t*-tests or Mann–Whitney U tests were used to compare differences in ABC scale scores between Tai Chi practitioners and non-practitioners within each cluster. Binary Logistic regression was applied to analyze the association between Tai Chi practice and fall history, calculating the Odds Ratio (OR) and its 95% Confidence Interval (CI).

## 3 Results

### 3.1 Correlation analysis of key variables

Pearson correlation analysis was used to explore the associations among the nine dimensions of sub-health, fall risk indicators, and Tai Chi exercise indicators (Table 1). Moderate positive correlations were observed between the various sub-health dimensions (*r* = 0.38 to 0.68, *p* < 0.01), indicating a synergistic relationship without severe multicollinearity. The ABC scale scores were significantly and positively correlated with all sub-health dimensions (*r* = 0.35 to 0.58, *p* < 0.01) and significantly and negatively correlated with the number of falls (*r* = −0.68, *p* < 0.01). The number of falls was significantly and negatively correlated with all sub-health dimensions (*r* = −0.28 to −0.53, *p* < 0.01), with the strongest correlations found for energy (*r* = −0.53) and motor function (*r* = −0.50). Regarding Tai Chi exercise indicators, frequency per week, duration per session, and years of practice were all significantly and positively correlated with ABC scale scores (*r* = 0.48 to 0.55, *p* < 0.01) and significantly and negatively correlated with the number of falls (*r* = −0.35 to −0.42, *p* < 0.01). These indicators also showed significant positive correlations with all sub-health dimensions (*r* = 0.22 to 0.44, *p* < 0.01), among which motor function exhibited stronger correlations with weekly frequency (*r* = 0.42) and duration per session (*r* = 0.44).

**Table 1:** Correlation analysis among sub-health dimensions, fall risk, and Tai Chi exercise indicators (*r* value, *n* = 612).

Variable	1	2	3	4	5	6	7	8	9	10	11	12	13	14
1. Physical symptoms	—													
2. Organ function	0.62**	—												
3. Motor function	0.58**	0.60**	—											
4. Energy	0.55**	0.58**	0.63**	—										
5. Positive emotions	0.48**	0.52**	0.49**	0.54**	—									
6. Psychological symptoms	0.52**	0.55**	0.53**	0.57**	0.68**	—								
7. Cognitive function	0.42**	0.45**	0.44**	0.47**	0.52**	0.55**	—							
8. Social adaptation	0.38**	0.41**	0.39**	0.42**	0.48**	0.46**	0.65**	—						
9. Social support	0.40**	0.44**	0.41**	0.43**	0.50**	0.51**	0.58**	0.62**	—					
10. Number of falls	0.48**	0.52**	0.55**	0.58**	0.45**	0.48**	0.40**	0.35**	0.42**	—				
11. ABC scale score	−0.42**	−0.45**	−0.50**	−0.53**	−0.38**	−0.41**	−0.32**	−0.28**	−0.35**	−0.68**	—			
12. Weekly frequency	0.35**	0.38**	0.42**	0.40**	0.32**	0.34**	0.28**	0.24**	0.30**	0.52**	−0.40**	—		
13. Duration per session	0.38**	0.40**	0.44**	0.42**	0.35**	0.36**	0.30**	0.26**	0.32**	0.55**	−0.42**	0.58**	—	
14. Years of practice	0.32**	0.35**	0.38**	0.36**	0.30**	0.31**	0.25**	0.22**	0.37**	0.48**	−0.35**	0.52**	0.55**	—

*p < 0.05, **p < 0.01.

These results indicate broad associations between sub-health status, fall risk, and Tai Chi exercise behavior among urban older adults, establishing a foundation for subsequent analyses.

### 3.2 Latent profile analysis of sub-health symptom clusters in urban older adults

Latent profile analysis was conducted on 612 urban older adults using the nine dimensions of sub-health as external indicators. The model fit indices are presented in Table 2. As the number of classes increased, the values of AIC, BIC, and aBIC continued to decrease. However, the LMR test results showed that the 2-class model (*p* = 0.0084) and the 3-class model (*p* = 0.0068) were both significantly superior to their preceding models. In contrast, the LMR tests for the 4-class (*p* = 0.3826) and 5-class models (*p* = 0.6311) were not significant, indicating no statistically meaningful improvement over the 3-class and 4-class models, respectively. Although the LMR test for the 6-class model was significant (*p* = 0.0166), the sample size of its smallest class was only 57 (9.3%), which approached the marginal threshold.

**Table 2:** Model fit indices for latent profile analysis of sub-health symptom clusters in urban older adults.

CLASS	AIC	BIC	aBIC	LMR (p)	BLRT (p)	Entropy	Grouping situation
1class	40482.870	40562.372	40505.225	—	—	—	612
2class	35700.637	35824.306	35735.411	0.0084	0.0000	0.959	217, 395
3class	32165.257	32333.092	32212.450	0.0068	0.0000	0.976	132, 269, 211
4class	30293.421	30505.424	30353.034	0.3826	0.0000	0.973	149, 152, 90, 221
5class	28555.468	28811.638	28627.501	0.6311	0.0000	0.979	74, 203, 110, 63, 162
6class	26977.529	27277.867	27061.981	0.0166	0.0000	0.984	64, 75, 117, 156, 143, 57

Considering the model fit indices, classification precision, and theoretical interpretability, the 3-class model was ultimately selected as the optimal model. The Entropy value for this model was 0.976, indicating exceptionally high classification accuracy. The sample sizes for each class were 132 (21.4%), 269 (43.5%), and 211 (34.1%), respectively, showing a reasonable distribution.

**Figure 2:**
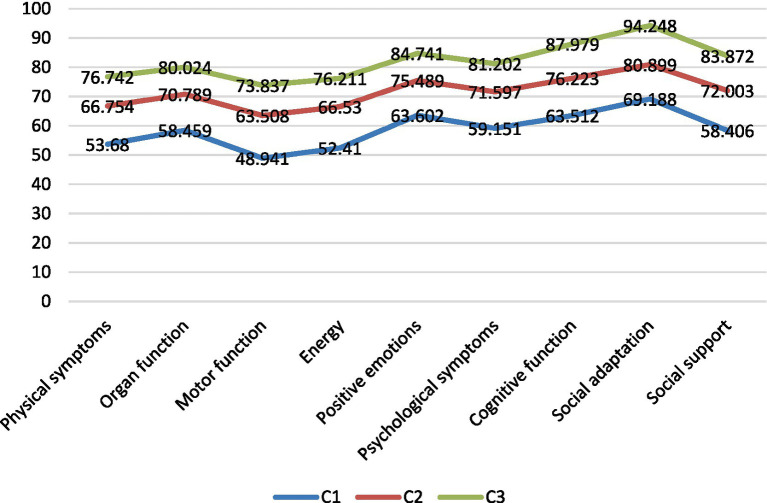
Latent profiles of sub-health symptom clusters among urban older adults.

Prior to the LPA, all 612 participants were screened to ensure that their total converted scores were below 60. The LPA identified three heterogeneous categories of sub-health symptom clusters among urban older adults (Figure 2, Table 3):

**Table 3:** Classification and characteristics of latent profiles for sub-health symptom clusters among urban older adults.

Profile label	Profile name	Defining characteristics	Sample size
C1	Low physical-psychological	Scores in physical and psychological health dimensions, such as motor function, energy, and psychological symptoms, are significantly low, indicating poor overall health status.	*n* = 132 (21.57%)
C2	Balanced average	Scores across all dimensions are relatively balanced without obvious deficiencies; this group represents the majority of the urban older adult population.	*n* = 269 (43.95%)
C3	Cognitive-social advantage	Scores in cognitive function and social adaptation are particularly outstanding; scores across all dimensions are generally high, indicating excellent overall health.	*n* = 211 (34.48%)

Low Physical-Psychological (C1, *n* = 132, 21.57%): This group scored significantly lower than the other two groups across dimensions such as motor function (48.94 ± 4.69), energy (52.41 ± 4.65), and psychological symptoms (59.15 ± 4.03). Their profile curve was positioned at the lowest level overall, with the most pronounced gaps in motor function and energy compared to C2 and C3. This group exhibited poor overall health and was located in the “high-risk zone” of sub-health, making them the primary target for health interventions.

Balanced Average (C2, *n* = 269, 43.95%): This group showed relatively balanced scores across the nine dimensions without obvious deficiencies. Their profile curve was positioned in the middle, maintaining a stable distance from C1 and C3. Representing the majority of the urban older adult population, this group is in the “gray area” of sub-health and requires active health management for maintenance.

Cognitive-Social Advantage (C3, *n* = 211, 34.48%): This group demonstrated particularly outstanding scores in cognitive function (88.00 ± 5.30) and social adaptation (94.26 ± 7.46). Their profile curve was at the highest overall position, with generally high scores across all dimensions. Characterized by excellent overall health status and situated in the “low-risk zone” of sub-health, this group serves as a typical representative of “successful aging.”

### 3.3 ANOVA results for the three latent profiles

The ANOVA results in Table 4 indicate highly significant differences across all nine sub-health dimensions among the three profiles (*p* < 0.001). The characteristics of each profile are as follows: Low Physical-Psychological (C1) scored the lowest across all dimensions. Specifically, scores in physical dimensions such as motor function (48.94 ± 4.69) and energy (52.41 ± 4.65) were both below 60 points, reflecting the coexistence of physical decline and psychological distress. Balanced Average (C2) exhibited scores at a moderate level, ranging from 63.51 to 80.95. This group showed relatively balanced health across dimensions and represents the majority of urban older adults. Cognitive-Social Advantage (C3) achieved the highest scores in all dimensions, with social adaptation (94.26 ± 7.46) and cognitive function (88.00 ± 5.30) being particularly outstanding, indicating excellent health status. The three profiles demonstrated a stable gradient distribution of C3 > C2 > C1. The score gap between C1 and C3 in motor function and energy reached approximately 24 points, suggesting that sub-health symptom clusters possess clear heterogeneous characteristics, which establishes a foundation for subsequent analyses.

**Table 4:** Comparison of sub-health dimension scores among urban older adults in different symptom clusters.

Variable	Profile 1 (*n* = 132)	Profile 2 (*n* = 269)	Profile 3 (*n* = 211)	*F*	*p*
Physical symptoms	53.68 ± 4.22	66.76 ± 3.14	76.77 ± 3.63	1711.206	<0.001
Organ function	58.46 ± 4.10	70.80 ± 2.82	80.05 ± 3.32	1738.977	<0.001
Motor function	48.94 ± 4.69	63.51 ± 3.25	73.87 ± 3.76	1773.223	<0.001
Energy	52.41 ± 4.65	66.54 ± 2.99	76.24 ± 3.44	1823.707	<0.001
Positive emotions	63.60 ± 3.79	75.50 ± 2.77	84.77 ± 3.26	1805.138	<0.001
Psychological symptoms	59.15 ± 4.03	71.61 ± 2.91	81.23 ± 3.37	1787.685	<0.001
Cognitive function	63.48 ± 5.45	76.26 ± 4.02	88.00 ± 5.30	1070.699	<0.001
Social adaptation	69.14 ± 6.81	80.95 ± 5.56	94.26 ± 7.46	622.538	<0.001
Social support	58.38 ± 5.54	72.03 ± 3.82	83.90 ± 5.11	1215.056	<0.001

Scores are presented as Mean ± standard deviation. C1, Low physical-psychological; C2, Balanced average; C3, Cognitive-social advantage. Dimensions were measured using the Sub-health measurement scale (SHMS V1.0). Post-hoc comparisons (Bonferroni): All dimensions followed the gradient of C3 > C2 > C1 (*p* < 0.05).

### 3.4 Multinomial logistic regression analysis of factors associated with sub-health symptom cluster membership

Multinomial Logistic regression analysis (Table 5) revealed that age, gender, education level, marital status, and monthly income were significant predictors of sub-health symptom cluster membership among urban older adults. Using Low Physical-Psychological (C1) as the reference group, the following results were observed: Comparison between Balanced Average (C2) and C1: Age (OR = 0.387), gender (OR = 0.043), education level (OR = 0.062), and marital status (OR = 0.054) were negatively associated with membership in C2, whereas monthly income (OR = 162.443) showed a positive association (all *p* < 0.01). Comparison between Cognitive-Social Advantage (C3) and C1: Age (OR = 0.275), gender (OR = 0.012), and marital status (OR = 0.139) were negatively associated with membership in C3, while monthly income (OR = 99.151) showed a positive association (*p* < 0.01). Education level did not reach statistical significance in this comparison (*p* = 0.303). Overall, younger, male, and higher-income older adults were more likely to belong to the Cognitive-Social Advantage (C3) group with superior health status. Conversely, those who were older, female, and had lower incomes were more likely to be categorized into the Low Physical-Psychological (C1) group with poorer health. The model demonstrated good fit (McFadden *R^2^* = 0.524, Nagelkerke *R^2^* = 0.762), suggesting the need to strengthen sub-health screening and precision interventions for advanced-age, female, and low-income populations, while addressing the differentiated health management needs of older adults with various demographic characteristics.

**Table 5:** Multinomial logistic regression analysis of factors associated with sub-health symptom cluster membership among urban older adults.

Variable	*β*	SE	Wald *χ*^2^	*p*-value	OR	95% CI
C2 vs. C1 (Ref: C1 low physical-psychological)						
Age	−0.948	0.245	15.030	<0.001	0.387	0.240 ~ 0.626
Gender	−3.157	1.095	8.314	0.004	0.043	0.005 ~ 0.364
Education level	−2.776	0.712	15.197	<0.001	0.062	0.015 ~ 0.252
Marital status	−2.912	0.682	18.247	<0.001	0.054	0.014 ~ 0.207
Monthly income	5.090	0.861	34.973	<0.001	162.443	30.062 ~ 877.768
C3 vs. C1 (Ref: C1 low physical-psychological)						
Age	−1.291	0.255	25.636	<0.001	0.275	0.167 ~ 0.453
Gender	−4.432	1.125	15.506	<0.001	0.012	0.001 ~ 0.108
Education level	−0.466	0.452	1.062	0.303	0.627	0.258 ~ 1.523①
Marital status	−1.970	0.635	9.622	0.002	0.139	0.040 ~ 0.484①
Monthly income	4.597	0.912	25.414	<0.001	99.151	16.584 ~ 592.713①

C1 is the reference group. ① indicates estimates adjusted by Firth’s penalized likelihood method. Model Fit, McFadden *R*^2^ = 0.524, Cox and Snell *R*^2^ = 0.671, Nagelkerke *R*^2^ = 0.762.

### 3.5 Comparison of demographic characteristics across sub-health symptom clusters

Chi-square tests were conducted to compare the demographic characteristics among the three latent profiles, with the results presented in Table 6. The analysis revealed significant differences across all demographic variables among the three profiles (*p* < 0.001). A clear trend of “rejuvenation” (younger age) was observed from Profile 1 to Profile 3 (proportion of those aged 70–74 years: 54.5% vs. 37.9% vs. 32.2%). Furthermore, moving from Profile 1 to Profile 3, several variables showed a gradual increase: The proportion of males (21.2% vs. 36.4% vs. 53.1%). Education levels (proportion of high school and above: 9.1% vs. 30.9% vs. 62.1%). Marriage rates (47.0% vs. 81.0% vs. 89.6%). Monthly income levels (proportion ≥ 4,000 RMB: 16.6% vs. 40.5% vs. 68.7%). Prevalence of overweight/obesity (34.9% vs. 39.4% vs. 66.8%). Smoking rates (10.6% vs. 21.2% vs. 34.6%) and alcohol consumption rates (15.2% vs. 28.6% vs. 42.2%). These findings indicate that the three latent profiles exhibit distinct demographic gradients: Profile 1 (Low Physical-Psychological) is characterized by advanced age, female gender, lower education levels, a higher proportion of widowed individuals, lower income, normal weight, and lower rates of smoking and drinking. Profile 3 (Cognitive-Social Advantage) is characterized by younger age, a higher proportion of males, higher education levels, being married, higher income, overweight/obesity, and higher rates of smoking and drinking. Profile 2 (Balanced Average) falls between the two for all mentioned characteristics.

**Table 6:** Comparison of demographic characteristics among urban older adults in different sub-health symptom clusters [*n* (%)].

Demographic variable	Label	Latent profile (%)			*χ*2	*p*
		1.0	2.0	3.0		
Age	60–64 years	5 (3.8)	38 (14.1)	42 (19.9)	189.452	<0.001
	65–69 years	28 (21.2)	98 (36.4)	89 (42.2)		
	70–74 years	72 (54.5)	102 (37.9)	68 (32.2)		
	≥75 years	27 (20.5)	31 (11.5)	12 (5.7)		
Gender	Male	28 (21.2)	98 (36.4)	112 (53.1)	42.683	<0.001
	Female	104 (78.8)	171 (63.6)	99 (46.9)		
Education level	Primary school or below	68 (51.5)	58 (21.6)	12 (5.7)	276.831	<0.001
	Junior high school	52 (39.4)	128 (47.6)	68 (32.2)		
	Senior high school/secondary specialized school	12 (9.1)	68 (25.3)	89 (42.2)		
	Junior college or above	0 (0.0)	15 (5.6)	42 (19.9)		
Marital status	Married	62 (47.0)	218 (81.0)	189 (89.6)	158.327	<0.001
	Widowed	58 (43.9)	42 (15.6)	18 (8.5)		
	Divorced/unmarried	12 (9.1)	9 (3.3)	4 (1.9)		
Monthly income	<2000 RMB	48 (36.4)	32 (11.9)	8 (3.8)	245.683	<0.001
	2000–3,999 RMB	62 (47.0)	128 (47.6)	58 (27.5)		
	4,000–5,999 RMB	18 (13.6)	82 (30.5)	98 (46.4)		
	≥6,000 RMB	4 (3.0)	27 (10.0)	47 (22.3)		
BMI	Underweight (< 18.5)	8 (6.1)	5 (1.9)	2 (0.9)	312.956	<0.001
	Normal weight (18.5–23.9)	78 (59.1)	158 (58.7)	68 (32.2)		
	Overweight (24.0–27.9)	38 (28.8)	86 (32.0)	112 (53.1)		
	Obese (≥28.0)	8 (6.1)	20 (7.4)	29 (13.7)		
Smoking	No	118 (89.4)	212 (78.8)	138 (65.4)	35.682	<0.001
	Yes	14 (10.6)	57 (21.2)	73 (34.6)		
Alcohol consumption	No	112 (84.8)	192 (71.4)	122 (57.8)	38.947	<0.001
	Yes	20 (15.2)	77 (28.6)	89 (42.2)		

### 3.6 Comparison of fall risk indicators across sub-health symptom clusters

Chi-square tests and ANOVA were conducted to compare the differences in fall risk indicators among the three latent profiles, with results presented in Table 7. The analysis revealed significant differences across all profiles in both fall rates and ABC scale scores (*p* < 0.001). From Profile 1 to Profile 3, the fall rate showed a gradual decrease (67.4% vs. 17.8% vs. 7.6%), while ABC scale scores showed a gradual increase (56.8 ± 9.6 vs. 77.3 ± 7.8 vs. 88.9 ± 6.5). These results indicate a significant gradient in fall risk across the three latent profiles: Low Physical-Psychological (Profile 1): Older adults in this group exhibited the highest fall risk and the lowest balance confidence. Balanced Average (Profile 2): This group occupied the middle ground for both indicators. Cognitive-Social Advantage (Profile 3): This group exhibited the lowest fall risk and the strongest balance confidence. These findings suggest that older adults in the Low Physical-Psychological group should be the primary target population for fall prevention interventions.

**Table 7:** Comparison of fall risk indicators among urban older adults in different sub-health symptom clusters [*n* (%)/M ± SD].

Variable	Profile 1	Profile 2	Profile 3	*χ*2	*p*
Fall rate in the past year	89 (67.4)	48 (17.8)	16 (7.6)	368.524	<0.001
ABC scale score (M ± SD)	56.8 ± 9.6	77.3 ± 7.8	88.9 ± 6.5	758.631	<0.001

### 3.7 Stratified analysis of the association between tai chi exercise and fall risk across sub-health symptom clusters

A stratified analysis was conducted to explore the association between Tai Chi exercise and fall risk within the three sub-health symptom clusters, with results presented in Table 8. The analysis revealed that within all three profiles, Tai Chi practitioners had significantly lower fall rates compared to non-practitioners (Profile 1: 12.2% vs. 92.3%, *OR* = 0.02; Profile 2: 3.8% vs. 48.8%, *OR* = 0.04; Profile 3: 1.1% vs. 60.9%, *OR* = 0.01; all *p* < 0.05). Additionally, ABC scale scores were significantly higher among Tai Chi practitioners than non-practitioners across all groups (mean differences of 14.6, 7.9, and 7.2, respectively; all *p* < 0.05).

**Table 8:** Stratified analysis of the association between Tai Chi exercise and fall risk indicators across sub-health symptom clusters.

Sub-health Profile	Tai Chi practitioners (*n*)	Non-practitioners (*n*)	Fall rate in Tai Chi practitioners [*n* (%)]	Fall rate in non-practitioners [*n* (%)]	OR (95% CI)	ABC score in Tai Chi practitioners	ABC score in non-practitioners	Mean difference (95% CI)
C1	41	91	5 (12.2)	84 (92.3)	0.02 (0.01–0.06)	67.8 ± 5.9	53.2 ± 8.1	14.6 (12.1–17.1)
C2	185	84	7 (3.8)	41 (48.8)	0.04 (0.02–0.10)	79.5 ± 4.8	71.6 ± 8.2	7.9 (6.2–9.6)
C3	188	23	2 (1.1)	14 (60.9)	0.01 (0.00–0.03)	89.6 ± 5.3	82.4 ± 8.9	7.2 (4.1–10.3)

These results indicate that Tai Chi exercise is significantly associated with reduced fall risk and enhanced balance confidence, and this protective effect is prevalent across different sub-health symptom clusters. Notably, the mean difference in ABC scale scores between Tai Chi practitioners and non-practitioners gradually decreased from Profile 1 to Profile 3 (14.6 → 7.9 → 7.2). This suggests that the effect of Tai Chi exercise on improving balance confidence is most pronounced among older adults with impaired health (Profile 1: Low Physical-Psychological).

## 4 Discussion

This study utilized latent profile analysis (LPA) to identify three heterogeneous sub-health symptom clusters among urban older adults: Low Physical-Psychological (C1), Balanced Average (C2), and Cognitive-Social Advantage (C3). Further analysis revealed significant differences across these clusters in terms of demographic characteristics, fall risk, and the strength of the association between Tai Chi exercise and fall risk. These findings provide an empirical basis for the precise identification of high-risk individuals and the development of individualized intervention strategies.

### 4.1 Heterogeneous characteristics of sub-health symptom clusters in urban older adults

This study found that sub-health status among urban older adults is not a monolithic structure but rather exhibits significant group heterogeneity. C1 (Low Physical-Psychological) scored significantly lower in dimensions such as motor function, energy, and psychological symptoms than the other groups. This suggests that this group experiences marked functional decline at both physiological and psychological levels, aligning with a “frailty-low mood” comorbidity pattern, making them the primary targets for sub-health intervention. C2 (Balanced Average) scores were at a moderate level, representing the health status of the majority of urban older adults. Although they have no obvious deficiencies, they also lack significant advantages, indicating a transitional state between health maintenance and decline. C3 (Cognitive-Social Advantage) achieved particularly outstanding scores in cognitive function and social adaptation, demonstrating a strong cognitive reserve and social support network, which reflects the typical characteristics of “successful aging (31).”

These results differ from previous variable-centered studies, which tend to treat older adults as a homogeneous group and overlook the combinatorial effects between symptoms. In contrast, the person-centered LPA approach allows for a more refined identification of the combination patterns of different symptom clusters, providing a classification basis for subsequent precision interventions (32).

### 4.2 Demographic differences across sub-health symptom clusters

The results from multinomial logistic regression and Chi-square tests indicate that age, gender, education level, marital status, and monthly income are critical factors influencing the membership of older adults in different symptom clusters. C1 (Low Physical-Psychological) is primarily characterized by advanced age, female gender, lower education, lower income, and being widowed (33). In contrast, C3 (Cognitive-Social Advantage) is predominantly composed of relatively younger, male, highly educated, higher-income, and married individuals. This gradient distribution highlights the pivotal role of structural social factors in the health differentiation of older adults (34). Notably, the prevalence of overweight/obesity, smoking, and alcohol consumption was significantly higher in C3 than in other groups, which appears to contradict their “health advantage” label. Potential explanations include: This group possesses superior economic conditions and engages in frequent social activities, leading to a more “unrestricted” lifestyle. The strong cognitive function and robust social support systems within this group may offset some of the negative impacts of these behavioral risks (35). This finding also suggests that having a health advantage does not equate to being free of behavioral risks. Future interventions should focus on maintaining their existing strengths while guiding them toward establishing healthier lifestyles.

### 4.3 Association between sub-health symptom clusters and fall risk

This study found a significant gradient in fall risk across the different symptom clusters. The fall rate for the C1 (Low Physical-Psychological) group during the past year was as high as 67.4%, while their ABC scale score was only 56.8—significantly lower than those of the C2 and C3 groups. These findings support the hypothesis that sub-health status serves as a critical precursor to fall risk (36). In particular, the strong correlation between scores in physiological dimensions (such as motor function and energy) and fall risk suggests that physiological decline is one of the core drivers of falls among older adults (37).

Although the C3 (Cognitive-Social Advantage) group exhibited excellent overall health, they still maintained a fall rate of 7.6% and a degree of decline in balance confidence. This indicates that even older adults categorized as “health-advantaged” face latent fall risks, highlighting that preventive interventions should not be overlooked even in high-functioning populations (38).

### 4.4 Variations in the association between tai chi exercise and fall risk across symptom clusters

This study further explored the strength of the association between Tai Chi exercise and fall risk within different symptom clusters. The results showed that across all three groups, Tai Chi practitioners had significantly lower fall rates and significantly higher ABC scale scores than non-practitioners, indicating that Tai Chi exerts a universal protective effect in reducing fall risk (9).

Notably, the mean difference in ABC scale scores between practitioners and non-practitioners was largest in the C1 (Low Physical-Psychological) group (14.6 points), and the disparity in fall rates was also most pronounced (12.2% vs. 92.3%). This suggests that older adults with poorer health status derive greater benefits from Tai Chi exercise (9). This may be attributed to their lower baseline balance confidence and greater potential for functional improvement (8). While Tai Chi remained protective for the C3 (Cognitive-Social Advantage) group, the magnitude of improvement was relatively smaller, reflecting a “ceiling effect” due to their already high health levels.

These findings carry significant public health implications: Tai Chi should not only be implemented as a universal fall prevention measure but should be prioritized for promotion among older adults with impaired health to maximize resource efficiency.

### 4.5 Innovations and practical implications of the study

The innovation of this study lies in being the first to combine latent profile analysis (LPA) with stratified analysis to identify heterogeneous patterns of sub-health symptoms among urban older adults. Furthermore, it systematically evaluates the variations in the association between Tai Chi exercise and fall risk across these distinct symptom clusters (39). These findings challenge “one-size-fits-all” health intervention models and emphasize the necessity of developing differentiated strategies based on the specific symptom profiles of older adults. From a practical perspective, the following recommendations are proposed: Sub-health Screening: Integrate sub-health screening into community health management processes for older adults to facilitate early identification of health status. Targeted Intervention for C1: Prioritize the identification of individuals in the Low Physical-Psychological (C1) group and recommend their participation in structured Tai Chi courses, as they derive the greatest benefit in balance confidence. Maintenance for C2: Encourage those in the Balanced Average (C2) group to maintain regular exercise habits to prevent further functional decline. Risk Awareness for C3: Although the Cognitive-Social Advantage (C3) group exhibits excellent health, efforts should still be made to enhance their risk awareness and prevent “health blind spots” related to latent fall risks (40).

## 5 Limitations and future directions

The present study has several limitations. First, the cross-sectional design limits causal inference. Specifically, a “healthy worker effect” (or healthy participant bias) cannot be excluded; older adults with better baseline health and functional capacity are more likely to engage in and adhere to long-term Tai Chi practice, which may lead to an overestimation of the exercise’s protective effects against falls. Second, although we conducted home visits to ensure diversity, the recruitment from community parks may introduce selection bias, as it tends to oversample active practitioners. Third, both Tai Chi practice habits and fall history relied on self-reports, which are susceptible to recall bias and social desirability bias. This might explain the high fall rate (92.3%) in the C1 non-practitioner group, as these frailer, homebound individuals may have clearer recall of their frequent fall incidents. Future research should utilize longitudinal designs and objective measures, such as wearable sensors for gait analysis, to mitigate these biases.

Additionally, while multiple dimensions of Tai Chi practice were collected, the grouping was reduced to a binary classification for stratified analysis. This simplification was necessary to ensure adequate statistical power within each latent profile, though it may result in some information loss regarding the dose–response relationship.

## 6 Conclusion

This study identified three heterogeneous sub-health patterns among urban older adults through latent profile analysis: Low Physical-Psychological (C1), Balanced Average (C2), and Cognitive-Social Advantage (C3). The findings reveal a significant gradient in fall risk across these profiles, with the C1 group exhibiting the highest risk and weakest balance confidence, identifying them as the core target population for intervention. Importantly, while Tai Chi exercise demonstrated a universal protective effect by reducing fall risk and enhancing balance confidence across all clusters, a distinct “compensatory effect” was observed: older adults with the poorest health status (C1) derived the greatest benefit, showing the most pronounced improvements compared to other groups. These results underscore the necessity of moving beyond “one-size-fits-all” approaches toward a precision prevention system. Consequently, it is recommended to integrate sub-health profiling into community health management for older adults, prioritizing the promotion of Tai Chi for health-impaired individuals to achieve strategic healthy aging goals.

## Data Availability

The original contributions presented in the study are included in the article/supplementary material, further inquiries can be directed to the corresponding author.
